# Corruption practices in drug prescribing in Vietnam – an analysis based on qualitative interviews

**DOI:** 10.1186/s12913-018-3384-3

**Published:** 2018-07-28

**Authors:** Tuan A. Nguyen, Rosemary Knight, Andrea Mant, Husna Razee, Geoffrey Brooks, Thu H. Dang, Elizabeth E. Roughead

**Affiliations:** 1 Quality Use of Medicines and Pharmacy Research Centre, Sansom Institute, School of Pharmacy and Medical Sciences, University of South Australia, GPO Box 2471, Adelaide, South Australia 5001 Australia; 2 School of Public Health and Community Medicine, University of New South Wales, Kensington, NSW 2052 Australia; 3 Pharmaceutical Consultant, Sydney, NSW 2025 Australia

**Keywords:** Developing countries, Ethics / moral perspectives, Health care professionals, Interviews, Semistructured, Medicine, Model building, Motivation, Qualitative analysis, Sensitive topics

## Abstract

**Background:**

Results from a previous study showed that 40 to 60% of the price of off-patent medicines in Vietnam was typically spent to induce prescribers to use the medicines, and to persuade procurement officers within hospitals to buy them. In this article we examine how and why inducements were paid by the pharmaceutical industry to health care providers in Vietnam.

**Methods:**

We use a theoretically informed analysis to understand pharmaceutical companies’ account of giving inducements and prescribers’ account of taking them, elicited through in-depth interviews.

**Results:**

Analysis of the emergent concepts derived from our qualitative data led to viewing the constructs from the theoretical framework of opportunities; pressures; and rationalization within a hierarchy of systemic factors and individual factors. Economic survival pressures in an imperfectly competitive market reportedly encouraged pharmaceutical companies and prescribers to be linked financially. Although individual factors such as professional ethics and personal values influenced doctors’ responses to corrupt practices, entrenched systemic issues, including lack of transparency, accountability, poor enforcement of legislation and prevalence of corruption emerged as important factors supporting corrupt practice or even making it very difficult for individuals to opt out of corrupt practices.

**Conclusions:**

Our theoretically informed analysis of inducements provides an in-depth understanding of an angle of corruption in Vietnam’s health sector, showing the need for multifaceted strategies in the fight against corruption in the health sector. Remedial strategies need to address both systemic and individual factors including interventions to relieve dependencies for survival of health care services on the corrupt system.

## Background

Corruption, the abuse of entrusted power for private gain [1], is a pervasive problem affecting many parts of the world. It is the single greatest obstacle to social and economic development [2] and one of the impediments to achievement of the Sustainable Development Goals [3]. In the health sector, corruption is a major threat to effective performance [4–7]. Within the pharmaceutical sector, among other negative impacts, corruption inflates pharmaceutical prices [8], wastes valuable resources allocated to pharmaceutical products and services [9], and is a major obstacle to strengthening pharmaceutical systems and increasing access to quality-assured medicines [10].

Recognising the detrimental consequence of corruption, international organizations including the World Health Organization (WHO) [11], the World Bank [12, 13], Medicines Transparency Alliance (MeTA) [14] and Transparency International [15] have begun to address corruption in the health sector and specifically corruption within the pharmaceutical sector. The first step is to identify where corruption occurs [9]. In the pharmaceutical chain, manufacturing, registration, medicine selection, procurement, distribution, prescribing and dispensing are all potential targets for corruption [16].

In late 2004, WHO initiated the Good Governance for Medicines Programme to reduce corruption in the pharmaceutical systems through the application of transparent, accountable administrative procedures and the promotion of ethical practices [11]. The first phase of this programme is the national assessment of the vulnerability to corruption of the national pharmaceutical system. The results of the assessments show that of the eight functions of the pharmaceutical system assessed, control of medicines promotion is the one most vulnerable to corruption. Medicines promotion focused on influencing decisions about medicine selection, procurement and prescribing, and marketing costs were a major contributor to the base price of medicines [17, 18].

In Vietnam, the 1986 economic reform process known as “Doi Moi” has led to important policy shifts in the healthcare system since 1989. A number of market-oriented measures were implemented, including the introduction of user fees at public health facilities, legalization of private pharmacy and medical practices, and liberalization of the production and sale of pharmaceuticals [19]. Vietnam’s near universal, publicly funded and provided health services were converted into an unregulated public-private mix [20]. The number of licensed private health facilities increased from virtually none in 1986, to around 65,000 in 2004, including 30,000 private health clinics, 23,000 private pharmaceutical units, and 12,000 traditional health units [21]. In 2008, 22 new private hospitals were licensed and constructed, in addition to the existing 74 private hospitals with 5600 beds. Private hospitals accounted for about 3% of the total number of hospital beds in Vietnam [22].

The opening of the country to foreign trade and the liberalization of rules governing pharmaceutical manufacture, sale and distribution transformed the Vietnam’s pharmaceutical supply chain from a centrally controlled system of few State-owned pharmaceutical enterprises to a market-oriented system with the participation of a vast array of companies. They included 180 domestic pharmaceutical manufacturers (including 22 Foreign Direct Investment - FDI producers), 90 importers, 800 domestic wholesalers/distributors, three FDI enterprises investing in drug logistics and 438 representative offices of foreign pharmaceutical companies by 2008 [23].

Vietnam’s pharmaceutical market was heavily dependent on imports. Imported medicines accounted for more than 50% of the market share, focusing on specialised products. Domestic medicine production accounted for an increasingly growing market share, rising from 36% in 2001 to approximately 50% in 2011, reaching USD 1.14 billion [24] but 90% of the raw materials used in domestic production were imported [25]. Nearly 95% of imported active pharmaceutical ingredients were antibiotics, vitamins, antipyretic, analgesics and anti-spasmodic drugs [26], reflecting a concentration of domestic pharmaceutical production on only some therapeutic classes. This led to a fierce competition due to trading duplication on some active substances, with paracetamol, for example, having 1044 registered products by 2011 [24]. The range of imported products was wider than those locally produced, however, trading duplication remained common, an example being 458 imported brands for one antibiotic cefixim by 2011 [24].

Vietnam’s transition from a socialist economy, to a market-based economy has presented a number of challenges for its past ‘human development’ achievements [27]. Increased reliance on market mechanisms has led to relative neglect of social mandates and a surge in health costs including medicine prices [21]. A previous survey on prices of 42 medicines in Vietnam found that medicine prices for patients in the public sector were 47 and 11 times higher than the international reference price (the median unit prices in the Management Sciences for Health Price Indicator Guide) for innovator brands and lowest-priced generic equivalents, respectively, after adjustment for purchasing power parity [28].

Main reasons for high medicine prices in Vietnam were subsequently investigated. A qualitative study initially interviewing representatives from two groups: pharmaceutical companies and private pharmacies who set their own medicine prices; and government officials responsible for controlling medicine prices in Vietnam was undertaken [8]. The authors found that 40 to 60% of the price of off-patent medicines in Vietnam was typically spent to induce prescribers to use the medicines, and to persuade procurement officers within hospitals to buy them. This led to a focus in more depth on the underlying causes of these inducements (a form of corruption), resulting in additional data collection.

In this paper, a theoretically informed analysis of the entire interview data is presented. Different theoretical frameworks have been developed to explain the structures that facilitate corruption, as well as the actors and motivators [29–35]. The theoretical framework of corruption in the health sector developed by Vian [7], which is based on economic principles and good governance, and integrates many earlier models and concepts of corruption including Cressey’s Fraud Triangle [36], was used to guide our coding and analysis. This framework classifies explanatory factors for corrupt behaviors into three main groups: opportunities, pressures, and rationalization [7]. Opportunities include monopoly, discretion, accountability, citizen voice, transparency and enforcement. Pressures include wages/incentives and pressure from clients. Rationalization includes social norms, moral/ethical beliefs, attitudes and personality [7].

## Method

### Participants and data collection

We have previously reported in detail the methods of the qualitative study examining the reasons for high medicine prices in Vietnam [8]. In brief, we initially recruited participants from the pharmaceutical industry and government officials responsible for controlling medicine prices in Vietnam using a combined method of purposive (c.f. [37]) and snowball sampling (c.f. [38, 39]). We used a loosely structured interview guide (c.f. [37]), asking participants to talk about how medicine prices were set in Vietnam and what components contributed to the final price of medicines, with a detailed account of expenses incurred as a medicine moved along Vietnam’s pharmaceutical supply chain.

As the study progressed, it became clear that participants considered inducements paid by pharmaceutical companies to prescribers and hospital procurement officers to be the main reason for high medicine prices in Vietnam. Consequently, we expanded the interview guide to examine the underlying causes of inducements. We also included a new group of participants (prescribers and hospital pharmacists) to hear their story about taking inducements. Using the same sampling techniques we purposely selected prescribers and hospital pharmacists according to a maximum diversification criterion [40] to capture a full range of views. Prescribers included those from both hospital consulting rooms (i.e. hospital outpatient clinics) and hospital inpatient wards, as well as those from all four major medical specialties: internal medicine; surgery; obstetrics and gynaecology; and paediatrics. Hospital pharmacists included both those from hospital pharmaceutical departments who provided pharmaceutical services to inpatients and those from hospital pharmacies who served outpatients. We recruited new participants from the two main hospital hubs in Vietnam: Hanoi in the North, and Ho Chi Minh City in the South.

The first author conducted all interviews after briefly describing the purpose of the study and obtaining participants’ written informed consent. Building rapport was undertaken with the introduction of the researcher’s ten-year experience working in the Vietnam pharmaceutical sector, including the pharmaceutical industry (pharmaceutical representative), pharmacy university (lecturer) and the government (drug appraisal specialist). This was essential to make the study informants feel less reticent given the sensitivity of the topics discussed. Each interview lasted between one and two hours and was audio-taped. When participants wanted to stop the tape because they talked about sensitive topics or did not want to be taped at all, then, with their agreement, only hand-written notes were taken.

Following the completion of each interview, *verbatim* transcription was undertaken. After reviewing the transcript several times to obtain a sense of the whole, a summary sheet of about one page was compiled (c.f. [41]), containing the main topics discussed in the interview and emerging themes to be further investigated and/or unanswered questions for the next contact. This preliminary analysis was conducted to guide the data collection process, with the emerging themes being addressed further in subsequent interviews. The combination of the summary sheet, the transcript, and field notes from the interview formed one interview record for final analysis.

### Data analysis

We used NVivo Version 8 software to code the interview records in Vietnamese. We did not use translated English versions because of the lack of equivalent words or phrases; there are words or concepts that cannot be translated from one language or cultural context to another, leading to distortion or loss of meaning [37, 42, 43]. In addition, even when linguistic equivalence is obtained, the nuance and intricacies of the original language are often lost in translation without consideration of the functional and cultural equivalence [44]. The literary devices (e.g. metaphors, analogies and proverbs), which are very much reliant on cultural context and difficult to translate, are often needed in qualitative analysis because of “their ability to bring richness, imagery and empathetic understanding to words” [45].

The first author completed the coding in collaboration with the other authors. We used Vian’s theoretical framework of corruption in the health sector [7] to develop a coding system. Specifically, we started building coding trees (a hierarchy of codes) with three broad categories of “opportunities for corruption”, “pressures for corruption” and “rationalization for corruption”. We grouped related codes under each category, based on conceptual relationships – “the same sort of things” [46]. We then further developed each category into sub-categories then sub-sub-categories, resulting in different coding trees. For example, the category “rationalization for corruption” was divided into sub-categories of “normalization of corruption” and “self-interest maximization”. Sub-category “normalization of corruption” was further divided into the sub-sub-categories “prevalence of corruption” and “other social norms”.

We applied a triangulation of researchers [37] in the development of the coding system. Two of the most information-rich interview records were translated into English with back-translation to consider conceptual equivalence and cultural appropriateness. The fourth author cross-coded the English version of these interview records to refine the coding system. Any differences in coding were re-examined and discussed among the research team until consensus was reached. The first author then used the refined coding system to code the rest of the interview records. The fourth author reviewed in full the initial coding of the first author following the English translation, with discrepant views resolved through team discussion. During the coding process, we created analytical notes using a project journal and memos. They were later used in the final stage of analysis, functioning also as an audit trail to document all decisions made to assist in maintaining theoretical rigour, as well as supporting evidence for the conclusions [47–49].

After classifying and sorting all codes into trees, we made connections across trees. We used constant comparison between categories across trees to find similarities and differences. We also examined the similarities and differences between individuals and groups to generate descriptions and patterns of association classified by categories at different levels. We used matrix coding queries and text search queries to assist in the identification of conditions that gave rise to each association pattern. We grouped together codes focusing around a common, broader concept, or were connected in a broader theme or theoretical relationship across trees using a “set” in the computer program QSR NVivo software [46]. We also used relationship nodes and models to generate these types of connections and to inform the synthesis of final conceptual frameworks.

## Results

### Participant characteristics

We conducted 43 in-depth interviews from April 2008 to December 2009 with 60 participants, including 37 individual interviews and six group interviews. Some respondents participated in both individual and group interviews. Two individual interviews with research participants from the pharmaceutical industry turned out to be group interviews as the primary participants came to the interview with other pharmaceutical industry representatives, who were also friends or used to be students of the first author. Four group interviews were also conducted with doctor participants instead of individual interview, following the suggestion of the primary participants. Two re-interviews occurred where confirmation of original material was required given the long period of data collection, which was due to the extensive documentation needed for this work. Twenty eight participants from the pharmaceutical industry, seven government officials, 21 prescribers and four hospital pharmacists contributed data. Themes emerged from data and their relationship are summarised in Table 1. Matrix of Intervening Factors for Corrupt Practices in Vietnam’s Health Sector.

**Table 1:** Matrix of intervening factors for corrupt practices in Vietnam’s health sector

	Systemic factors	Individual factors
Opportunities for corruption	Poor governance	None
	Discretion	
	Transparency	
	Accountability	
	Enforcement	
Pressures for corruption	Pharmaceutical market related factors	Pharmaceutical market related factors
	Product related factors	
	Regulation related factors	
	Survival in the market (Sales-based compensation policies)	Survival in the market (Pressure to achieve sale targets, fear of being fired if targets are not achieved)
	Health care structures and processes	
	The tender system	
	Remuneration systems and financial pressure	
	Workplace pressures	
Rationalization for corruption	Normalization of corruption	Self-interest maximization
	The prevalence of corruption	Professional ethics
	Other social norms	Personal values
		Knowledge and skills
		Advancement opportunity
		Reputation
		Employment

### Opportunity for corruption

#### Poor governance

Participants suggested that opportunities for inducements in the Vietnam health care sector were mainly related to poor governance, which included: (a) the degree of personal discretion afforded participants; (b) lack of transparency; (c) lack of accountability in administrative procedures; and (d) a low probability of detection and follow-up enforcement.

##### Discretion

Discretion refers to the autonomous power of officials to make decisions [35]. Participants reported a number of areas in Vietnam’s health sector where discretion was exercised improperly, including medicine tendering practices in public hospitals and prescribing practices. In relation to tenders, pharmaceutical company participants noted that there were arbitrary selection specifications for contractors supplying medicines to public hospitals. Tender committees often organized discriminatory terms and conditions which favoured particular tenderers as reflected below:

*Hospitals require that you [pharmaceutical companies] must have had a history of [at least] three years selling the products to the hospitals [where they want to bid] to be able to go to the next round of tendering, so although your price is cheaper, you are still kicked out.*. *.*. *They don’t even let you attend the tendering. If you cannot attend the tendering, then when will you get three years of experience?*. *.*. *Hospitals set a lot of hurdles and virtual barriers for tendering so they can use them to fail companies that don’t have “good relationships” with them although the prices of these companies are cheaper. (A private pharmaceutical company manager in an individual in-depth interview).*

Discretion was also portrayed by the concept of *“a low level of democracy”* [individual decision making versus multidisciplinary decision making] when participants talked about provincial hospitals. In most provincial hospitals, pharmaceutical department heads had autonomous power in making decisions on medicine procurement and were able to influence prescribing of particular medicines in their hospitals. This was because of a lack of *“KOLs [key opinion leaders]”* among prescribers at the provincial level. It is different from central hospitals where there were prescribers who were a national leader in their specialty and often made requests to procure the medicines of their choice. Therefore, in provincial hospitals pharmaceutical companies often focused on inducing the heads of hospital pharmaceutical departments with higher kickbacks:

*The kickback to hospital pharmaceutical department ranges from 3 to 15% [of the sale]. It depends. With pharmaceutical departments of provincial hospitals it can be up to 30%.*. *.*. *In provinces with a low level of democracy where the pharmaceutical department can “eat” [take] all [because] doctors have no voice, it can be 30–40% [of the sale] and doctors have nothing or just a little. (A private pharmaceutical company manager in a group interview).*

With prescribers, discretion was manifested by their freedom to prescribe medicines. Pharmaceutical company participants reported that in hospital outpatient consulting rooms, prescribers had full freedom to prescribe specific medicines for outpatients. By contrast, in hospital inpatient wards, prescribed medicines were limited to the hospital formulary list and the availability of medicines in hospital pharmaceutical departments. Therefore, prescribing medicines in return for commissions was reported to be more prevalent in consulting rooms than in inpatient wards. The commission for prescribers in consulting rooms was, therefore, also often higher. A private pharmaceutical company manager remarked: *“Prescribers in consulting rooms have more [higher commission rate], often up to 65 per cent [of the sale].*. *.*. *The commission for prescribers in inpatient wards is only around 15-35 per cent.”* Information on *“who prescribed what and how many”* was often recorded and provided to pharmaceutical companies by a nurse in each inpatient ward in exchange of a monthly salary of USD 10 to 15. In consulting rooms, pharmaceutical companies relied on information provided by hospital pharmacies to double-check the prescribing information self-provided by their *“contracted”* prescribers (also called collaborators). Participants from private pharmaceutical companies reported that staff in many hospital pharmacies ran a service of counting prescriptions for pharmaceutical companies with a fee of around *“USD 10 per product per month.”* However, outpatients had their freedom in buying medicines either from hospital pharmacies or private pharmacies outside the hospitals so the pharmaceutical companies still had to pay a commission to their prescribers according to the number of prescriptions in their prescribers’ self-record which was often higher than that provided by hospital pharmacies.

Doctor participants pointed out that where freedom of prescribing was limited, such as for doctors in central and teaching hospitals, prescribers often had to think twice before prescribing because their behavior was always being observed by their students. However, when prescribers were free to prescribe whatever they wanted without adequate controls, they were more likely to abuse their discretionary power for private gain. One doctor participant from a teaching hospital confirmed that *“in other hospitals [not teaching hospitals] people [prescribers] often do that [prescribing for commission].”*

##### Transparency

Effective transparency allows stakeholders to access information on not only the basic facts and figures, but also the mechanisms and processes [1]. Disclosure of information on how decisions are made and performance measures helps to reduce the discretionary power of government officials and reinforce accountability. Lack of transparency, however, was widely considered to be *“one of the weakest points in governance in Vietnam”* both in the content of regulations and in the implementation of the regulations in the health sector.

Sometimes, the regulations were said to lack transparency because the regulations contained ambiguous provisions that enabled the authorities to act at their own discretion, which caused difficulty for pharmaceutical companies wanting to comply with the rules. Both a private pharmaceutical company manager and a private pharmacy owner spoke of how they felt threatened unless they were extra welcoming to the authorities: *“When they come, if you don’t treat them well, you will “die” [be in big trouble]”* because *“with [the ambiguity of] our regulations, anywhere the authorities look [inspect], they will find alleged breaches of regulations [breaches resulted from alternative interpretations of the ambiguous regulations by the authorities].”*

Pharmaceutical company participants often spoke of a lack of transparency in implementation of tender regulations, with committees not publicly revealing the opening bid prices. This allowed some tenderers to subsequently manipulate their bid prices. A private pharmaceutical company manager remarked: *“Who will be successful in tender is known beforehand because those [pharmaceutical companies] who have bribed tender committees are informed of our prices, and then they change their price accordingly.”*

Lack of transparency was also reported in Vietnam’s financial system, where most transactions were undertaken on a cash-only basis. A manager from a State-owned pharmaceutical company commented: *“In Vietnam, all financial activities are done with cash-in-hand so we cannot control corrupt practices. We need to do transactions through bank accounts. In an economy without a transparent financial system, we cannot control corrupt practices.”*

Participants also reported that increasing transparency would prevent corrupt practices. For example, in hospitals where electronic software was used to undertake tendering, they said that all tender specifications were transparent and that tender dossiers were assessed by the same computerized rules, rather than at the discretion of individuals. This strategy reduced lobbying pressure and the need for inducements for the tender committees. However, participants indicated that few hospitals had introduced the tender software.

##### Accountability

According to Brinkerhoff [33], accountability refers to the obligation of individuals or agencies to inform other actors about their decisions and actions, to justify or explain them, and to suffer sanctions and punishment for non-performance, misconduct or corrupt behaviors. Whereas the first two components of accountability (i.e. providing information on what was done and why) form answerability, which is the essence of accountability, the expected magnitude of any applicable punishment and sanctions for misconduct “gives teeth to accountability” [33].

Participants stated that poor answerability in Vietnam’s health sector created more autonomous prescriber power, encouraging prescribers to order unnecessary medicines for their private gain as reflected below:

*I know there is a doctor who prescribes four tonics in one prescription. That is because patients don’t know what they are for. If patients know about this, how can the doctor prescribe like this? We don’t have to explain what medicines are used for what purposes so patients still have to buy all medicines prescribed. If it is explained that these are four similar tonics, patients are surely not going to buy them all. (An internal medicine doctor in a group interview).*

Although asymmetries in information and expertise are characteristic of all health services [33], exploiting them is a manifestation of poor answerability. Doctor participants admitted exploiting such asymmetries as reflected in the following quote:

*When working in [hospital] consulting rooms, we divide patients into two groups. Those from rural areas don’t know much then you can do that [prescribe perceivably low quality medicines to have high commission]. The second group is*. *.*. *[name of dwellers of a city] and they have more than enough knowledge to recognize which medicine is from Europe.*. *.*. *Therefore, with city patients don’t be silly to prescribe those medicines [low quality medicines with high commission]. (A paediatrician in a group interview).*

Health insurance funds serving as the agents of individual patients often play an important role in holding health care providers accountable [33]. However, as revealed in a group interview with doctors, in Vietnam, the public health insurer was considered not to have fulfilled this function, making answerability poor. That was reportedly because the public health insurance is operated by the Vietnam Social Security – an outsider of the health sector from physicians’ perspective, whereas it is the Ministry of Health to have direct power over health care providers.

In some cases where answerability did exist, participants asserted that the punishment and sanctions for misconduct were ineffectual. For example, referring to the fine for breaching pricing regulations, one participant said that *“a fine of [VND-Vietnamese currency] 300,000 [equivalent to USD 12.5] is not enough to even threaten a kid in year three.”* This led to *“sanction resistance”* as reflected below:

*Our sanctions are often not strict enough. For example, with the regulation of 30% CIF price [the price that includes Cost, Insurance and Freight to bring the product to the port of destination], companies are willing to be fined to break it. Accepting the fine makes sense because the profit we get by breaking the regulation is much more than the fine. (A private pharmaceutical company manager in an individual in-depth interview).*

Similarly, doctor participants thought that the sanctions for misconduct in public hospitals were not strong enough to deter prescribers’ inappropriate behavior and actions. In many public hospitals, when collusion between prescribers and pharmaceutical companies was detected, the corrupt doctors were just *“given verbal warnings”* or, at worst, had to *“move from one department to another”.* Dismissal was most frequently reported by doctor participants as a symbol of strong sanctions. One doctor participant emphasized that *“losing a job will lead to a series of problems,”* thus suggesting that Vietnam needed to *“have stricter sanctions to make the laws powerful.”*

##### Enforcement

Brinkerhoff [33] states that “sanctions without enforcement significantly diminish accountability.” Enforcement relies on the probability of detection of corrupt acts and the probability that punishment will follow detection. To enforce accountability, detection and monitoring systems are needed to collect evidence on corrupt practices. However, these systems were reported to be generally inadequate in Vietnam’s public health care sector.

Detection and monitoring systems varied. Participants reported that in Ho Chi Minh City, hospitals generally had better detection systems than in Hanoi, and inpatient treatment wards had better detection and monitoring systems than in hospital consulting rooms. The existence of these monitoring systems acted as a deterrent to some extent:

*It [prescribing for commission] happens mostly in [hospital] consulting rooms, because in the inpatient treatment wards what medicine is prescribed is clearly recorded in the patient chart so they [prescribers] are afraid. In consulting rooms, patients just take the prescription then leave buying their medicines outside [private pharmacies or hospital pharmacies] so there is no record of what is prescribed. (A manager from a foreign company in an individual in-depth interview).*

When corrupt practices are detected, the enforcement mechanisms are only effective if those who engage in such practices are punished strictly, demonstrating the need for accountability and responsiveness to government agencies [33]. Enforcement needs to be associated with strict sanctions, regulated in broad legal frameworks by institutions capable of enforcing them. Doctor participants said that in Vietnam *“the laws do exist but the enforcement is poor.”* We identified two main reasons for poor enforcement.

The first reason was insufficient institutional capacity in the public sector. At the health facility level, prescriber participants were concerned about the inability of those in power to punish the guilty. They said that *“if found to be corrupt, whether they [corrupt people] will be punished depends on a number of factors.”* This was reportedly because the public health facilities were not independent and were often influenced by authorities at higher levels in the public system, who had the power over the management board of the facilities. By contrast, private facilities were relatively more independent and the management board had their full power to run the facilities for the best interest of the hospital owners. Doctor participants who were employees of a public hospital but also worked after-hour for a private clinic said that when they worked in the private sector, they did not dare to prescribe medicines for private gain because they would be sacked immediately by their clinic managers. Organizational mechanisms in the private sector seemed to empower the directors of private hospitals or clinics, allowing them to have direct control over hospital rules and policies, and decisions on dismissal of their staff as reflected below:

*The pre-eminence of my hospital over public hospitals is that at the beginning of the establishment, the management board has laid down as a policy the development of a model hospital without informal payments. For example, two nurses were sacked because of being caught behaving corruptly. We are unanimous throughout the hospital [about the policy] so that all staff are afraid of breaching our regulation. If they are caught [being corrupt] they will be sacked. (A chief pharmacist of a private hospital in an individual in-depth interview).*

The second reason for poor enforcement in Vietnam’s public health sector was the apparent lack of commitment to action by management boards. A consensus among all participant groups was that department heads or directors of public health facilities all knew that their staff were taking inducements from pharmaceutical companies but little was done to address the problem. This was because *“they [the management board members] might also engage in corrupt practices for private gain so they can’t give lessons to others.”* A head of a clinical department remarked: *“It takes time. We cannot do anything just overnight. The main concern is if there is someone wanting to solve this [corruption] or not. They all benefit from this [corruption] so nobody will deprive their benefit themselves.”*

Overall, lack of enforcement or selective enforcement undermines trust in the fight against corruption. A consensus among participants was that they did not think the government could combat corrupt practices. Most participants believed that *“at least in the next 10 years, Vietnam is still not able to address the corrupt practices in the health sector”* because they were so convinced of the lack of any consequence following corrupt practices. A pharmaceutical company manager said: *“We dare to tell you all our secrets because when you are able to address the problem [corrupt practices in the health sector], our hair will have turned white. This problem has existed for many years.”*

### Pressures for corruption

Participants’ responses revealed two salient sub-categories of pressures for corrupt practices. They were related either to the pharmaceutical market or to health care processes and structures.

#### Pharmaceutical market related factors

Participants spoke of typical features of the pharmaceutical market that placed pressures on companies to offer inducements including products, regulations and market survival.

##### Product related factors

Special requirements for pharmaceutical registration, including the requirement of an expiry date, were reported by pharmaceutical industry participants as placing a heavy pressure on them to sell their medicines prior to their expiry date:

*Medicines have been imported. If they are not sold out, they will expire in the store then the company will “die” [fail]. Therefore, the more collaborators [doctors who receive monthly payment from pharmaceutical companies to prescribe the company’s medicines] the better, even then the profit left [for the company] is very small, and the money mainly goes into the pocket of collaborators. The company still has to go ahead. Otherwise, they have to throw their medicines away*. *.*. *so they have to sell at all costs. (A manager from a foreign company in an individual in-depth interview).*

Low quality, efficacy and reputation of a medicine were also mentioned as placing pressure on pharmaceutical companies to resort to inducements. For example, doctors were more easily persuaded to prescribe innovator brands and generic medicines from Europe as these were believed to be highly reputable, of high quality and efficacy. By contrast, Asian generic medicines traded by domestic companies were perceived to be lower in quality and efficacy and therefore, companies resorted to strategies such as *“buyout”* to create incentives for prescribers. With this strategy *“all the needs of a prescriber such as a car, money and so forth were satisfied.”* In return, the prescriber had to work for these pharmaceutical companies, as remarked below:

*In my hospital, there is a doctor who was the head of a clinical department. After investigating we found that there was evidence of collusion between her and a pharmaceutical company because she prescribed too frequently a medicine of this company. We decided to transfer her to another department, but she insisted on staying there for a while to use up the medicines because she had signed a form of contract [with the pharmaceutical company]. She took a big amount of money from the company then she had to use a certain quantity [of this medicine] in a certain period. (An internal medicine doctor in a group interview).*

Pharmaceutical industry participants reported that initially, Asian sourced medicines were preferred because of high commissions (normally 30–40% of the sale but can be up to 65%). However, after a while, many patients treated with these medicines did not get better. For patients with severe or recurrent illness, prescribers had to choose better European products instead. This was why European medicines with lower commissions (reportedly about 15%) were still able to be sold.

##### Regulation related factors

Pharmaceutical industry participants reported that Vietnam’s medicine pricing authorities used an unwritten rule in appraising the pharmaceutical registration dossier of companies before granting a marketing authorization for a medicine. If the difference between the declared selling price and the declared buying price [often CIF price – the price that includes Cost, Insurance and Freight to bring the product to the port of destination] of the medicine was more than 30% of the CIF price, the registrant of the medicine would be requested to provide a rationale. If not forthcoming, the medicine would be unlikely to be granted a marketing authorization.

**Fig. 1:**
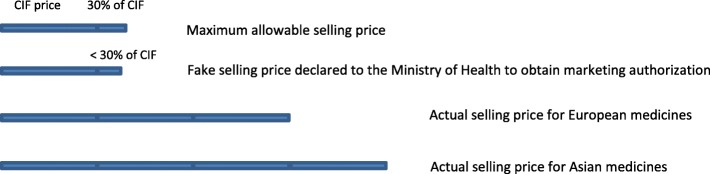
The manipulation of the pharmaceutical selling price

Private pharmaceutical companies set a selling price for their medicines that was several times the CIF price (2.5 to 3 times for European medicines and 3.5 to 4 times for Asian products). Consequently, pharmaceutical companies faced the problem of not being able to have a marketing authorization from the Vietnam regulatory authorities. Pharmaceutical companies seemed to solve this problem either by declaring a lower selling price (Fig. 1) or a higher buying price as reflected below:

*So we keep violating the regulation. If the Ministry of Health catches us, we will pay a fine but we still violate the regulation. We declare a fake, low selling price to get a “visa number” [marketing authorization]. Then when we’ve got the visa, the real price will be adjusted depending on the market. (A private pharmaceutical company manager in an individual in-depth interview)*.

**Fig. 2:**
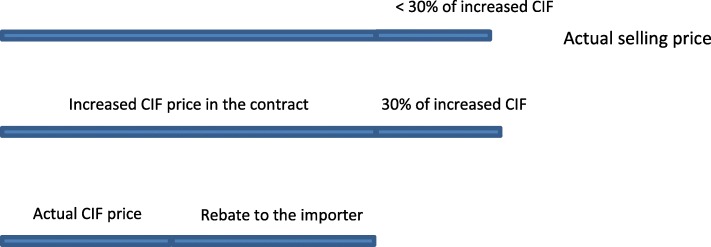
The manipulation of the pharmaceutical buying price by increasing the actual CIF price

Pharmaceutical companies reported manipulating the buying price up to a level that was about 30% lower than their planned selling price. Participants reported two strategies enabling this. The first was asking the foreign exporter to increase the contract price. Pharmaceutical importers then paid the fake, increased price according to the contract with a subsequent rebate provided (Fig. 2), and thus opening them to the risk of losing their money in case the exporter did not provide the rebate. Because of reportedly strict laws against *“money laundering”* in Western Europe and North America, this approach was almost impossible when the manufacturers were from these regions. Rather, it was used with generic manufacturers from *“India, Korea, some countries in Eastern Europe and South America.”*

**Fig. 3:**
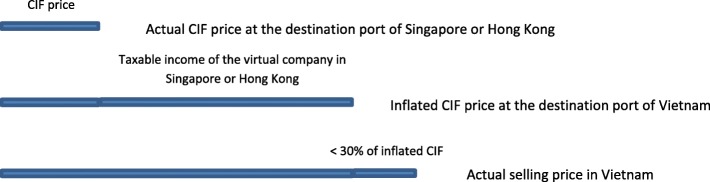
The manipulation of the pharmaceutical buying price by using a virtual company overseas

The second strategy, used more often, was that Vietnamese pharmaceutical companies established a *“virtual company in countries with low rates of corporate tax such as Singapore or Hong Kong.”* Medicines from foreign manufacturers were reportedly exported to the virtual company with the real buying price. From the virtual company, the price of medicines was inflated up to a level that was close to the planned selling price in Vietnam, and then exported to Vietnam. Pharmaceutical companies were then able to get marketing authorization for their medicines (because the gross profit was less than 30% of the inflated CIF price), selling at a high price in Vietnam, but by paying a low corporate income tax in Singapore or Hong Kong (Fig. 3).

Participants noted that establishment of a virtual company overseas was costly. Only big companies with great financial potential were able to manipulate the buying price by themselves. Other companies had to hire big companies to do so, and then pay them a price manipulation fee of *“four per cent of the difference between the real price and the manipulated price.”*

##### Survival in the market

Pricing authority participants from the Ministry of Health believed that increases in pharmaceutical supply as a result of increased number of companies would *“help to stabilize medicine prices.”* However, pharmaceutical industry participants claimed that the demand and supply theory was not applicable to Vietnam’s pharmaceutical market, especially for prescription-only medicines. Instead of reducing prices to attract more demand as with normal commodities, pharmaceutical suppliers increased their prices to fund increased inducements to prescribers, so as to maintain or increase their market share. This was because *“the utmost aim of every company is to sell their medicines in the market and gain profit to survive.”*

Participants pointed out that prior to the health sector reforms in 1989, when only some State-owned pharmaceutical enterprises produced and supplied medicines to the domestic market, there was virtually no competition among pharmaceutical companies:

*Initially, doctors did not have that behavior [taking commission from pharmaceutical companies]. In the time of the subsidy period* 1 *there had been nobody thanking by cash. . . . We did not have many medicines, there was no competition. Only a few medicines were available and we automatically prescribed them. (A head of a clinical department in an individual in-depth interview).*

Following the health sector reforms, the introduction of foreign companies and domestic private companies to the market created significant competition. The setting of a high enough price or mark-up, to fund the payment of commissions to ensure adequate market share, became a vital issue for domestic pharmaceutical companies. Compliance with the regulations also worked against company survival. Participants said that, to survive, companies had no choice but to violate the law:

*My company complies with regulations, setting prices with a mark-up of only 30% [of] the CIF price. Other companies don’t comply with regulations. They set their prices with higher mark-up, and then their products are sold faster because their commissions and kickbacks are higher.*. *.*. *So we also have to break the regulations to be able to sell our medicines. (A private pharmaceutical company manager in an individual in-depth interview).*

With increasing competition in more recent years, companies seemed to resort to additional strategies such as using sale-based compensation policy to motivate sale staff or paying key opinion leaders, who were chiefs and played a decisive role in procurement and use of medicines in the hospital. According to doctor participants, key opinion leaders were given *“gifts by way of stocks,”* becoming significant shareholders of the companies. Apart from the value of the stocks given, key opinion leaders received annual dividends from the companies concerned.

Fierce competition among domestic pharmaceutical companies also forged alliances with different companies who dealt with different products that therapeutically complement one another in a certain treatment. As an alliance of several companies, the conglomerate would be able to *“buyout”* the targeted prescribers. In this case, the prescriber was *“tối đa hóa [maximized]”* to serve as a prescribing machine for the conglomerate. Participants suggested that prescribers of this kind were often those in consulting rooms of big hospitals and they prescribed according to a standard *“formula”* that contained exactly the same four to five medicines for almost every patient.

There seemed to be a consensus among participants that multinational pharmaceutical companies tended to prohibit bribes. Officially at least, they did not have a commission policy at a global level for prescribers to address the issue of competition. However, pressure to achieve sales targets and the fear of being sacked if not achieving their assigned targets often meant that pharmaceutical representatives ignored global policy of their multinational company and gave money directly to prescribers. Such money was reported often not to be in the form of a percentage of the sale paid to prescribers, as was the normal practice of private domestic pharmaceutical companies. Rather, it was considered as *“gratitude to prescribers for their support.”* Nevertheless, the 2010 state media investigation revealed that there were multinational pharmaceutical companies that used commission of up to 30% of the sale to induce prescribers [50]. At other times, prescriber participants reported that multinational pharmaceutical companies paid for one-off benefits like *“a luxury holiday package”* as an incentive for *“using their products until the sales reached a certain amount,”* which was essentially the same as buyout strategies applied by some Asian and domestic traders.

Because the inducements were illegal and not tax-deductible, they were often legalised by pharmaceutical companies to be under the name of legitimate marketing costs to avoid paying a higher corporate tax. For example, they could be indicated as the expenses for key prescribers to attend domestic and international conferences (airfare, accommodation) but in fact the expenses were for luxury holiday packages that included a conference. Multinational companies also had samples – not for sale to provide to prescribers for free trial in their patients. However, these samples, after receiving by pharmaceutical representatives, were reportedly often converted into money, gift, etc. to induce prescribers. With domestic pharmaceutical companies, inducements were also hidden under fake input business expenses such as fake input invoices or salary of a virtual sale force through declaring a *“higher number of staff.”* These were tax evasion and came at a price however, as pharmaceutical companies had to bribe tax officials. A pharmaceutical company manager admitted: *“They [tax officials] know that we evade corporate tax.*. *.*. *To ensure you pay a low tax, you have to lobby them. As an unwritten law, you will keep two thirds and your tax official will get one third of the amount you evaded.”*

#### Health care processes and structures

The following salient factors related to health care processes and structures that placed pressures on pharmaceutical companies to resort to inducements, and on prescribers to take these inducements were generated from participant responses: (a) the tender system; (b) the remuneration system; and (c) workplace pressures.

##### The tender system

For a tender to be eligible for consideration, the active substance of a medicine had to be listed on the public health insurance reimbursement schedule issued by the Ministry of Health. According to pharmaceutical company participants, they had to *“lobby to put medicines on the list [reimbursement schedule] of the Ministry of Health,”* and this involved providing inducements to authorities. By contrast, authority officials felt unduly pressured when they applied objective criteria in listing or de-listing particular medicines from the schedule as reflected below:

*A representative of*. *.*. *[name of a company] came here.*. *.*. *I knew in the gift bag there was cash. I told her “I know what you say but all scientific evidence proves that your medicine is not effective.” . .*. *She threatened me that in her company there were people whose parent was this person, that person [high ranking authorities who can negatively influence the participant’s position].*. *.*. *They used*. *.*. *[name of a professional association] to put pressure on the Ministry of Health, they came to my house to lobby me and when they failed they threatened me.*. *.*. *I was much stressed. Other people who have my position might kiếm chác [earn] a great deal but I don’t.*. *.*. *Some medicines [with unclear effects] have been de-listed but still there are many that tons of cash are being paid for. (An official from the Ministry of Health in an individual in-depth interview).*

Based on the reimbursement schedule of the Ministry of Health, each hospital developed its own formulary list as a basis for tendering and prescribing medicines. The hospital formulary tender list often comprised of two lists, an innovator brand name medicine list and a generic list. Whereas the trade name of the medicine was specified in advance when tendering for innovator brand medicines, tendering for generic medicines only specified the active substance in advance, thus allowing more competition in the tender. Being on the innovator brand medicine list of the tender was of great advantage to suppliers:

*In my company, expense for tender lobby is limited: only about USD 250–300 per product. But it is for putting the product on the brand name list only. To be successful in the tender is a different story. I’ve heard there are products, especially Korean and Indian ones, of which the expense is up to USD 1000 per product.*. *.*. *By lobbying to be on the brand name list, then the medicine will be our own. The price will be brand name prices, thus being higher than on the generic list. (A pharmaceutical representative from a private company in a group interview).*

Lobbying in the tender processes was reported to be so common and necessary that many companies had to establish a *“separate division specializing in lobbying for tenders.”* More recently, many hospitals have used tendering systems to procure medicines, not only for insured patients, but also to serve un-insured outpatients in hospital pharmacies. Thus, the pressure to be successful tenderers has become greater, creating more incentives for pharmaceutical companies to lobby tender committees more forcefully. When *“everybody pays money to lobby tender, the amount spent for tender lobby increasingly grows.”* In key markets, pharmaceutical companies were willing to *“bribe [influential people] with a Camry [a Toyota car] to be successful in tender.”*

##### Remuneration systems and financial pressure

An inadequate level of remuneration was the most frequently reported factor leading prescribers to engage in corrupt practices. Often, participants spoke of inadequate salaries that were insufficient for prescribers to satisfy their essential needs, especially those who had just graduated. Low wages was what participants believed led some prescribers to take up inducements as a way of augmenting their income. An internal medicine doctor participant admitted that currently *“the main income of doctors in internal medicine is from pharmaceutical companies, by way of commission.”* Another participant remarked:

*Only for food it costs them [prescribers] almost USD 100 per month, whereas their monthly salary is just USD 100, how can they raise their children, how will they deal with when they are sick, how can they buy their house?*. *.*. *We need to pay them properly, and then the corrupt practices will reduce. (A private pharmaceutical company manager in an individual in-depth interview).*

The low wages, however, did not seem problematic with physicians from some other specialties such as surgeons, who reportedly had other sources of income such as informal payments from patients when they delivered the operation. An obstetrical surgeon participant said that *“In fact, I don’t need commission. I don’t know about others but with me money has never been a matter. I have never cared about commission.”*

Participants also spoke of financial pressures in a broader way. This referred to the pressure of having money not only to satisfy their basic needs, such as food and clothes, but also higher ranking needs such as further education or holidays and travel. Even the rich were said to experience financial pressure to maintain their life style. This was said to explain why many well-off prescribers still colluded with pharmaceutical companies:

*Everybody needs stability. It means that even when they are earning a lot they still worry. Last month they earned USD 5000 and were living in a rank of people earning USD 5000; playing golf with a cost of thousands of dollars, all of a sudden their income reduces by half, then they are not able to play golf any longer; feeling disappointed immediately so they have pressure. The poor have their pressure but the rich have their pressure too, just different types. (A doctor from an intensive care unit in a group interview).*

Another theme that emerged was unfairness in prescriber remuneration. Doctor participants complained that their salary did not correspond to their responsibility and qualifications. To be a doctor, they reported that they had to be very intelligent students to enter medical schools and their university training was longer than for other professions. Upon graduation, they had to work harder and had more responsibilities, dealing with life and death decisions. Yet, their salary was ranked *“at 17*^*th*^ *among 19 professions paid directly by the government.”* The salary of a public doctor with 20 years’ experience was reported to be less than the salary of *“an engineer who had just graduated working in an IT [information technology] or banking area.”* The remuneration for the health care service of clinicians was compared with that of labor work such as *“pumping up a tyre in a street”* or *“a cleaner,”* leading to discontent particularly among more junior doctors. A doctor explained how the unfairness in remuneration led to some of them resorting to corruption:

*I think unfairness causes discontent. People look out and recognize that this guy doesn’t do anything, but still earns a lot of money. He spends freely without thinking, going out with his wife and children all days then buying a beautiful modern car whereas we stick with our old bicycle, lacking food. His children have been sent to famous schools overseas whereas our children have to study in “lởm khởm” [low quality] local schools.*. *. . Finally, we feel that that’s not OK. We think to ourselves, oh dear he can take [money from corruption] so we can take too. Why don’t we take it? If we don’t take it, it will be taken by others. (A head of a clinical department in an individual in-depth interview).*

Financial disparity was reported to be even more important than a low salary in explaining corrupt practices. A doctor said that *“in the past the salary was very low too”* but when the whole society was poor, financial pressure did not automatically lead to corrupt practices.

##### Workplace pressures

Doctor participants reported that in their daily work, varied pressure from their managers, colleagues or subordinates could also influence their prescribing behaviors and response to corrupt practices. For example, one doctor talked about the heavy pressure coming from his departmental head:

*In fact, I don’t need commission. I don’t know about others but with me money has never been a matter. I have never cared about commission but there are a lot of influences. For example, my boss tells me that you are only allowed to use this medicine. Who dares not to listen to him? You don’t want to listen, right? Stop operation. Now working in a surgery department and your name is not on the operation schedule, can you imagine? Your boss just puts your name off the list in two weeks then you will do whatever he tells you to do. There are factors that make us have to follow [engage in corrupt practices] although we don’t want to. (An obstetrician in a group interview).*

Some doctors used the metaphor *“turning on the green light”* to indicate that they received an encouraging signal from their managers to use a certain medicine after their department was financially sponsored by a company. Many other participants stated that although pressure from managers did exist, it was just mild pressure. There were also doctors from internal medicine who said that they *“have never had any pressure from bosses.”*

Doctor participants also pointed out that sometimes they had pressure from their subordinates to engage in corrupt practices. A departmental head said that she had a policy that her department would only use high quality medicines from multinational pharmaceutical companies. However, she found it hard to explain to her staff that there was no commission when prescribing these medicines in the context where *“other pharmaceutical companies gave doctors too much.”* Finally, to reconcile this situation, she had to ask companies whose medicines were being used in her department to pay *“a little commission of USD 0.25 instead of USD 0.75 [per vial]”* or set aside an amount to *“buy a gift for my staff.”*

### Rationalization of corruption

Participants also spoke of socio-cultural factors and individual beliefs and attitudes that helped people to normalize and justify corrupt practices. The following sections discuss the two most salient sub-categories: (a) the normalization of corruption; and (b) self-interest maximization.

#### The normalization of corruption

Corrupt practices in Vietnam were reported to be so prevalent that all participants considered it inevitable and embedded in Vietnamese society. In addition, the Vietnamese cultures where people often express their gratitude to those supporting them also reportedly contribute to the normalization of corrupt practices.

##### The prevalence of corrupt practices

Doctor participants used the prevalence of corruption in Vietnam as an excuse for their corrupt practices as noted below:

*It’s not only the health sector being corrupt, but it’s the whole society, every sector. The higher ranking, the worse people are. You should know that when corrupt practices become normal, incorrupt people become the minority and in the society’s eyes, they become abnormal. Thus, how can you tell us to stay outside? (An internal medicine doctor in a group interview).*

Corrupt practices seemed pervasive. The recruitment in the health care sector was no exception:

*I think in the near future, we cannot solve the problem [of corrupt practices in the health sector]. Simply put, it is because they [doctors] have to buy their position in hospitals with a lot of money. They have just graduated. How they can have much money if they don’t take money from pharmaceutical companies or patients? (A private pharmaceutical company manager in an individual in-depth interview).*

Those who engaged in corrupt practices, particularly junior doctors, rationalized their behavior stating that *“abuses come from the chiefs”* and *“before being in management positions, they [the chiefs] all did the same as we are doing now [engage in corrupt practices],”* and that their corrupt practices was *“nothing compared with that of big people [high ranking people] and it is big people who hold sinecures; they don’t do much but grab all manna [Live on the fat of the land - ngồi mát ăn bát vàng].”*

According to participants, in a society where informal payments were necessary to get things done, even people who did not want to engage in corrupt practices still had to do so. Therefore, *“it is impossible for the health sector to be incorrupt in a corrupt society.”* Pharmaceutical industry participants said they *“have to adapt to the given market, although they want to do business with integrity,”* whereas doctor participants justified that *“in this context [corrupt society], it is doctors who are influenced by the society rather than they are the cause of the problem.”*

##### Other social norms

The pragmatism of mutual support seemed to reinforce the normalizing of corruption. Pharmaceutical company participants said that giving commissions to prescribers was *“a normal thing”* because *“thanks to their [doctors’] contribution, our company can develop.”* Therefore, *“when the company has profit, our customers [the doctors] have to have profit too.”* This logic was also used by doctor participants as reflected below:

*I like prescribing the medicines of those who often come to see me. To be honest, everybody will be like that. We will prescribe medicines with so-so quality but have commission for us. Everybody is like that. Those who often come to visit us, taking care of us, to be honest we will pay more attention as a way of thanking them. (A paediatrician in a group interview).*

#### Self-interest maximization

Using the economic cost-benefit consideration, doctor participants rationalized or explained corrupt practices by indicating a number of individual factors that influenced their responses to corrupt practices. Doctor participants reported that, before deciding whether or not to engage in an improper behavior, they had to consider the expected consequences of their actions. They weighed the costs and benefits of engaging in the improper behavior versus not engaging, and opted to maximize their self-interest. Doctor participants said that whereas the benefit of colluding with pharmaceutical companies was the financial gain from corrupt practices, the cost was a risk of losing their *“assets.”* These assets included (a) professional ethics; (b) personal values; (c) knowledge and skills; (d) advancement opportunity; (e) reputation; and especially (f) employment.

##### Professional ethics

Professional ethics were reported by doctor participants to be a factor preventing them from engaging in corrupt practices as remarked below:

*With me patients are always first. It’s not because they [pharmaceutical companies] give me more or less then I prescribe more for them. It depends on the severity of patients. With severe patients, I still have to use good quality medicines of big [multinational innovator brand] companies even if they don’t have commission at all, I still have to. This is principle. (A paediatrician in a group interview).*

Participants acknowledged that the ethical degradation of the whole society, together with the various pressures of life, eroded professional ethical standards. Medical ethics weakened when financial gain from corrupt practices *“increased to a level that was rewarding enough.”* A doctor participant suggested that it was more important to *“create an institutional mechanism that does not allow people to engage in misconduct, even when their professional ethics weaken”* rather than to try to improve professional ethics.

There was also confusion over what was deemed to be ethical behavior. Some doctor participants considered it was ethically acceptable to take offers from pharmaceutical companies (e.g. precious gifts, holiday packages and even commissions) as long as the offer was given without their asking for it, or after their recommendation of a particular medicine:

*They [pharmaceutical companies] come and give me cash every month. They said that “you have used this quantity [of products] so I give you this amount [of money].” However, I don’t know the commission accounting for how many percent [of the sale] and I don’t know if I have prescribed many or few, and I don’t care about this. I only know that I have selected their medicines and they come to thank me. (An internal medicine doctor in a group interview).*

Another example of medical ethics confusion was that doctors considered medicines as a normal commodity. Because they were *“customers of pharmaceutical companies,”* taking gifts or commissions from pharmaceutical companies was seen to be similar to a customer receiving a promotional gift, a normal act in every type of trading in Vietnam. Some doctor participants believed that it was morally acceptable if commissions were for the collective rather than individual interests, such as sponsoring a holiday package for all members of a clinical department.

##### Personal values

In addition to professional ethics, other moral norms such as *“conscience”* and *“dignity”* were also reported to prevent people from engaging in corrupt practices. Participants stated that an important determinant of whether a person was morally better than others was his or her good family tradition:

*I think family tradition is very important. Why I have never been bought over? In the past, my grandfather and then my father used their own money to exchange for our freedom.*. *.*. *They exchanged their entire asset for freedom [talking about the participant’s family who dedicated their entire asset to the Vietnam revolution in August 1945 to have the country’s independence].*. *.*. *Why now I have to exchange my freedom for some pence. Although I am not rich, I can’t do this. To be honest, many people nowadays sell their freedom too cheap. (An official from the Ministry of Health in an individual in-depth interview).*

##### Knowledge and skills

Most doctor participants believed that prescribers’ knowledge and expertise influenced their behavior and responses to corrupt practices. A common view was that the greater the expertise, the less the susceptibility to corrupt practices. An internal medicine doctor remarked *“people with good expertise often don’t prescribe for commission. They are less influenced by medical representatives, whereas those with low expertise often prescribe for their own interest.”* Some doctor participants indicated that individual personality or lifestyle preference and needs were more important than knowledge in influencing behavior.

##### Advancement opportunity

Another asset that was often reported by doctor participants as hindering them from engaging in corrupt practices was their advancement opportunity. Participants stated that some people did not dare to engage in corrupt practices because they wanted to keep themselves *“clean”* to have opportunities for advancement in the future. When asked at what age doctors were more likely to prescribe medicines for a commission, a common answer was that both old and young doctors were equally vulnerable. However, the older doctors, who held certain positions in hospitals, were reported to be more likely to engage in collusion with pharmaceutical companies than the younger ones because *“young doctors have a long future ahead.”* If they were caught being corrupt, *“their future would be negatively affected.”* Nevertheless, those junior doctors who had to *“buy”* their position in hospitals were reportedly at a high risk of engaging in corrupt practice to make up the amount of money they spent to have their job.

##### Reputation

Reputation was also an asset that doctors could lose if they prescribed poor quality medicines for private gain. A doctor participant said *“when working in our private clinic, we don’t care about the commission from pharmaceuticals. We have to do the best thing for patients, selecting the best medicines. Our treatment has to be successful; otherwise patients will not come back.”* In the private sector, building high reputation to attract patients appeared to be a more sustainable option for doctors to generate income than taking inducements then loosing patients. This was because poor quality providers were eliminated as patients selected higher quality providers. By contrast, such automatically enforced accountability was reported not to exist in the public hospitals which were *“always over-crowded and prescribers still received their government salary regardless of their performance.”*

##### Employment

The risk of losing one’s employment was another individual deterrent to engaging in corrupt practices. All participant groups argued that the higher the salary, the less likely that the doctors would engage in corrupt practices to risk their well-paid job. A pharmaceutical company participant illustrated: *“For example, in*. *.*. *hospital [name of a foreign-invested hospital] you can’t induce doctors with commission to have medicines prescribed because doctors in this hospital are very well paid so they are very scared of being sacked if they do the wrong thing.”*

### A hierarchy of systemic factors and individual factors

**Fig. 4:**
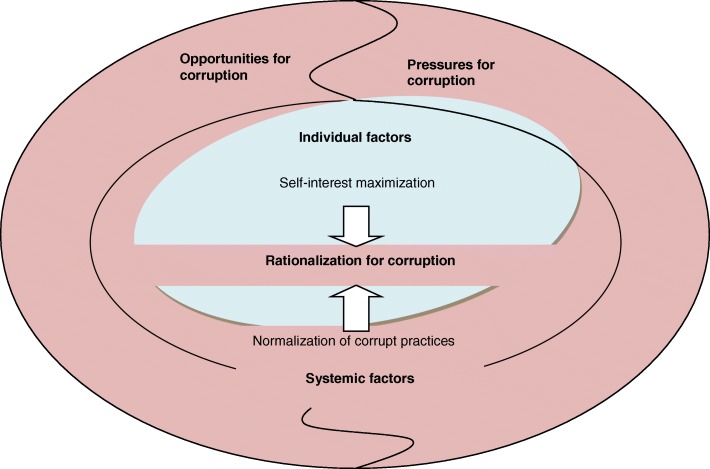
An extended theoretical framework of corruption in Vietnam’s health sector

Our constant comparisons of codes between categories led us to viewing the constructs from the theoretical framework of opportunities, pressures, and rationalization within a hierarchy of systemic factors and individual factors (see Fig. 4. An extended theoretical framework of corruption in Vietnam’s health sector).

**Fig. 5:**
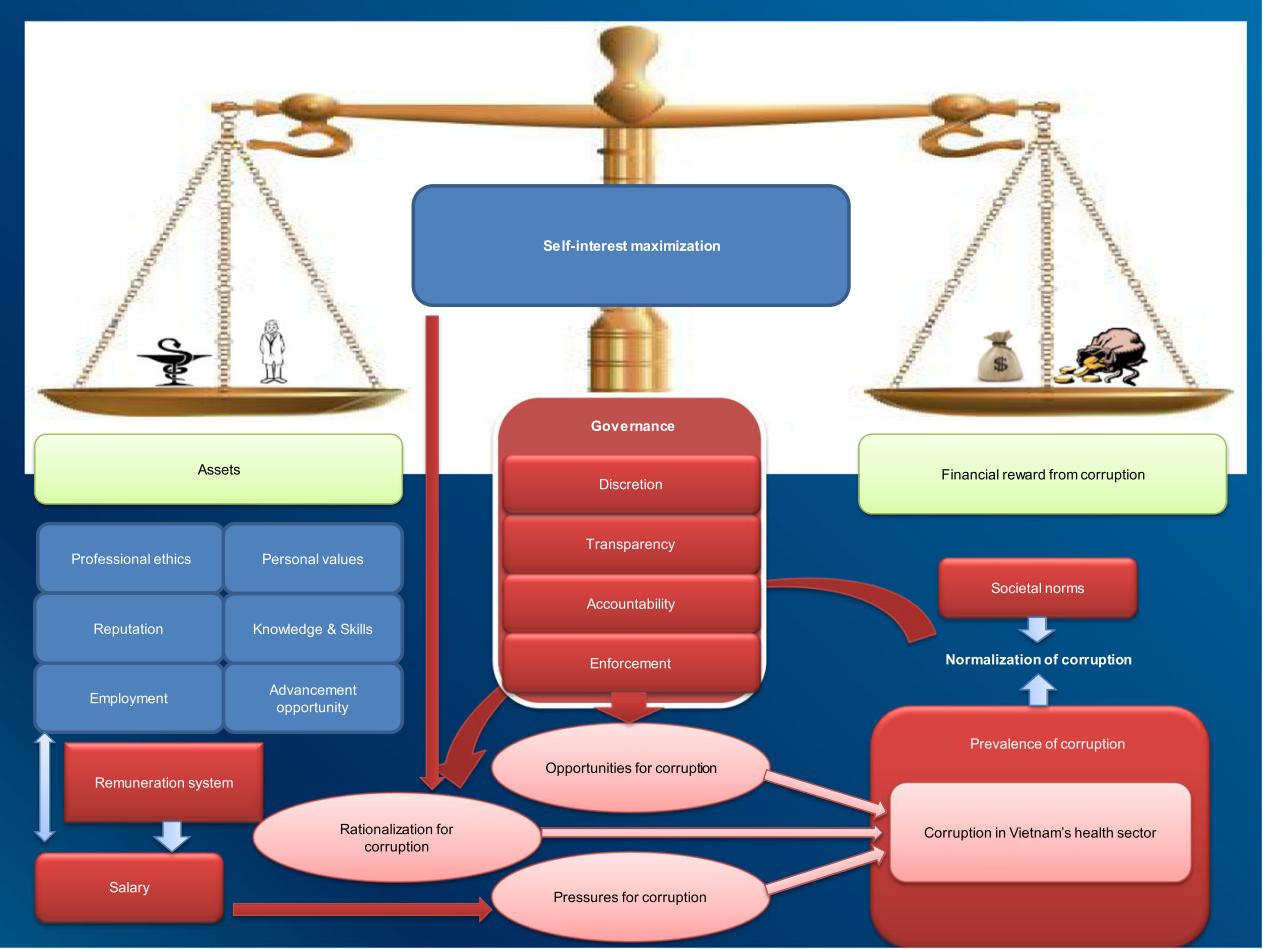
The Trade-off model explaining corrupt behaviors in Vietnam’s health sector

In this extended framework, systemic factors, which comprise opportunities for corruption, systemic pressures for corruption and normalization of corruption, act as a necessary condition facilitating corruption. Individual factors which include self-interest maximization from economic theory [51] and individual pressures provide a sufficient condition for corrupt practices. The dominant corrupt behaviors discussed in this article (i.e. the collusion between prescribers and pharmaceutical companies) can be conceptualized in the form of a Trade-off model (see Fig. 5. The Trade-off model explaining corrupt behaviors in Vietnam’s health sector) that represents the interaction between the individual and systemic factors. The evidence gained from our study suggests that broken systems (systems that create structural opportunities, or that create incentives for abuse or don’t protect and encourage people who want to act with integrity) can make it easier for individuals to engage in corruption. The systems may even make it very difficult for people to opt out of acting in ways that they know are morally wrong or abusive.

Among individual factors, the salient theme of self-interest maximization is at the centre of the model (Fig. 5). The dominant theme of governance among systemic factors plays a role at the base of the scale, making people face the trade-off between financial gain from corrupt practices and their assets. If governance systems are poor, people do not have to choose. They can gain financially from corruption without worrying about losing their assets. Rather, corruption gains will be added to their assets, making their total assets bigger, satisfying their self-interest maximization. However, this interaction between self-interest maximization and governance, which creates individuals’ rational choice, is strongly pre-determined by another dominant theme of prevalence of corruption among systemic factors. Individuals in highly corrupted systems may not have choices because as reflected by a doctor participant *“It’s not only the health sector being corrupt, but it’s the whole society, every sector.*. *. . incorrupt people become the minority and in the society’s eyes, they become abnormal. Thus, how can you tell us to stay outside?”*

A doctor participant used an interesting metaphor which illustrates the applicability of this model to the situation of corruption in Vietnam’s health sector. He said,

*It‘s like a tiger. When the tiger is not hungry, it doesn’t have any need. People are the same. When they don’t have to worry about money, they don’t have a need to earn money. To do so, we need an appropriate direct remuneration system. On the other hand, we need a functioning monitoring system. Although the tiger is full, we still need an electric stick and an iron fence; otherwise it can jump out at any time. People are similar, human beings are good in principle but when they have the opportunity, they are easily corruptible in the absence of penalties. (A head of a surgical department in an individual in-depth interview).*

## Discussion

The results of our study show that participants believed that economic survival pressures, in an imperfectly competitive market, facilitated pharmaceutical companies and doctors to be linked financially. Although individual factors such as professional ethics and personal values influenced doctors’ response to corrupt practices, entrenched or intractable systemic issues, including lack of transparency and accountability and poor legislation enforcement, emerged as important factors perpetuating corruption. More importantly, the systemic factor of prevalence of corruption in Vietnam society predetermined individuals’ choices, making them difficult to opt out of corrupt practices. The magnitude of reported corrupt practices varied across geographic regions, sectors, and doctors’ specialties.

To understand why and how inducements occur, the perspectives of both the givers and recipients of inducements were considered in this analysis. The givers were invariably the pharmaceutical companies. However, the recipients were reported to include authorities in the health sector, in tax offices and in market management departments, doctors and drug procurement officers.

Analysing the inducements made by pharmaceutical companies that contributed to the inflated medicine prices in Vietnam, we found a number of areas where corruption often occurred in the health and pharmaceutical sectors. These included key pharmaceutical system decision points such as drug selection (to be on the public health insurance reimbursement list, the hospital formulary list or the innovator brand name medicine list for tendering), procurement (kickbacks for tender committees), distribution (from pharmaceutical companies to private pharmacies with fake invoices, establishment of virtual companies overseas to evade taxes) and prescribing (commissions to prescribers). This finding confirms previous work that has identified vulnerable decision points for corruption in the pharmaceutical system worldwide [9, 16].

Our findings are also supported by other work in Vietnam. Pharmaceutical companies providing commissions to prescribers based on prescribing history has been documented and believed to contribute to the irrational use of medicines in Vietnam [4]. A United Nations Development Program funded study examined media reporting on health sector corruption from five national-level Vietnamese media outlets between 2008 and 2009 and found that 18% of the stories reported were about gaining commissions from the sale of medicines [52]. The perceptions of the prevalence of corruption in society, people’s pessimism about combating corruption and evidence of weak enforcement and monitoring have also been reported as factors contributing to corruption in the health sector in other work [53–55].

We used the theoretical framework of corruption in the health sector developed by Vian [7] to frame the reasons for corrupt practices in Vietnam’s health care sector elicited by participant responses. Vian’s framework consists of three main categories: opportunities; pressures; and rationalization. Analysis of the emergent concepts derived from our qualitative data led to the additional development of an extended theoretical framework of corruption, comprising systemic factors and individual factors to explain corrupt practices.

This extended theoretical model helps to explain the varying engagement in corruption by individuals in a society. The systemic factors might be the same in one society or one organisation, however, the difference in individual factors lead to behavioural differences and different responses to corrupt practices. That is why participants unanimously agreed that those doctors with a good family tradition, expertise, high reputation, good opportunity for advancement in the future, or those in foreign direct investment hospitals where salaries were very high, were less likely to engage in corrupt practices for private gain.

Health-related corruption is complex, difficult to detect and hard to treat [56]. While distinguishing the individual and systemic factors for corruption, our proposed model also shows the interconnectedness between them. On the one hand, it makes it easier to tackle corruption. The anti-corruption programs based on our model can be designed in such a way that helps to deal with each component of the corruption model. This is similar to breaking individual chopsticks being much easier than breaking a whole bunch of those chopsticks. On the other hand, it shows the necessity of a holistic approach for effective interventions to combat health-related corruption through addressing both individual and systemic factors at their different stages. For example, educational interventions targeting moral beliefs might work in those who have confusion over what is deemed to be ethical behaviour. They are, however, less likely to work in many others who already know corrupt behaviour is bad but believe that there will be no consequences following corrupt practices. A similar observation was noted by Vian and Burak (2006) when their study participants thought that informal payments were bad, but important [57].

Our theoretical model is likely to be applicable to corrupt practices in the health sector in many other transition economies and could be used to guide assessments of corruption in those countries. As in Vietnam, the rapid deregulation and decentralisation of the pharmaceutical sector, combined with an unstable economic environment in those countries not only created opportunities to engage in corruption but was also a survival strategy for many low salary health workers [16, 58]. Not everyone succumbed to the pressure to engage in corrupt practices, but it was reported to be quite prevalent in Vietnam. In addition, individual factors (such as scant ethical beliefs, personality attributes) together with some systemic factors (including the prevalence of corruption in society and societal norms) helped some (but not all) people justify their corrupt behaviors. Confusion over what was deemed to be ethical behavior have also been reported to contribute to corruption in Vietnam, a similar situation in other transition economies [31].

Our study has some limitations. First, according to Lee and Renzetti (1990), research which ‘potentially poses for those involved a substantial threat’ is regarded as sensitive research [59]. Research may be considered threatening if it poses an intrusive threat for participants because it deals with deeply personal issues that are private, stressful or fearful; it impinges on the interests of those being studied; or it studies deviance or social control [59]. Medicine prices are often regarded as trade secrets [60] and high medicine prices have been a concern in the public domain in Vietnam [61]. Studying the corrupt practice that leads to high medicine prices in Vietnam not only intrudes into the private sphere of pharmaceutical companies, health professionals and employees, but also deals with issues of social control that impinge strongly on the interests of the people being studied. Given these features, this study is considered to be highly sensitive research with high potential for reticent issues as participants might have been influenced by social desirability bias. A number of strategies, however, have been employed to reduce the reticence including building rapport by the researcher, stopping recorder or not using recorder at all when participants requested so, and using snowball sampling technique.

Second, while use of the snowball sampling technique facilitated the identification of participants for sensitive research, it might have resulted in groups of likeminded participants. Third, the data were collected during the period from April 2008 to December 2009, and the situation in Vietnam has since changed, although our proposed model is probably still valid. The long duration of data collection made us have to use re-interviews to confirm the initial interviews and original data but member checking was not undertaken for every study participant. Nevertheless, a number of strategies for validity was employed. They include researcher triangulation (in coding process), participant triangulation (data were collected from both the inducement givers and takers in diverse professionals, specialties and settings), and use of analytical notes and memos as audit trails. Also included was negative/deviant case analysis (searching for and discussing elements of the data that do not support or appear to contradict the main patterns or explanations emerging from data analysis). The iterative process of data collection and analysis where the preliminary analyses through the summary sheets was used to guide the subsequent data collection, as well as theoretically informed analysis were also employed to improve the validity of our interpretation of the data. As Hammersley and Atkinson (1995) suggested, efforts to triangulate data, method, researcher, and theory to bolster accuracy and reliability are all convergent with the inherent logic of transactional validity in qualitative research [62].

## Conclusion

The three groups of factors, namely the opportunity for corruption, pressure for corruption and rationalization of corruption, moderated by the economic theory of self-interest maximization and trade-off decisions, together explained many of the corrupt practices of giving and taking inducements in the health sector, which participants said contributed strongly to high medicine prices in Vietnam. Our theoretically informed analysis of inducements provides an in-depth understanding of an angle of corruption in Vietnam’s health sector, showing the need for multifaceted strategies in the fight against corruption in the health sector. Remedial strategies need to address both systemic and individual factors including interventions to relieve dependencies for survival of health care services on the corrupt system.

This paper therefore has potential impact on the fight against corruption in the Vietnam health sector. Practically, it has identified where corruption occurs, the first essential step to combat corruption. Theoretically, it has proposed an adapted model to explain the varying engagement in corruption by individuals in the Vietnam health sector, thus not only contributing to the body of knowledge but also helping the design of interventions and the development of strategies in tackling different factors that are necessary and sufficient to lead to corruption in the health sector.

## Abbreviations

CIF: Cost Insurance and Freight
USD: US dollar

## Footnotes

1 The period prior to ‘Doi moi’, an economy reform of Vietnam in1986, was followed by the health sector reforms in 1989.
